# Artificial Intelligence in Cardiology: Applications in Diagnosis and Risk Prediction

**DOI:** 10.7759/cureus.112841

**Published:** 2026-07-17

**Authors:** Anand Sekar G, Ajit V Kulkarni, Chetan Kumar Sharma, Bansari Yagnik Tank, Sharat Vishwanath K, Hairya Ajaykumar Lakhani

**Affiliations:** 1 Department of Cardiology, Aarupadai Veedu Medical College, Vinayaka Missions Research Foundation (VMRF-DU), Kirumampakkam, IND; 2 Department of Medicine, Navodaya Medical College Hospital and Research Centre, Raichur, IND; 3 Department of Community Medicine, Virendra Kumar Sakhlecha Government Medical College, Neemuch, IND; 4 Department of Microbiology, Dr. N. D. Desai Faculty of Medical Science and Research, Dharmsinh Desai University, Nadiad, IND; 5 Department of Mental Health Nursing, Indian Council of Medical Research (ICMR) and Bhopal Memorial Hospital and Research Centre (BMHRC) Bhopal Nursing College, Bhopal, IND; 6 Department of Medicine, Smt. B. K. Shah Medical Institute and Research Centre, Vadodara, IND

**Keywords:** artificial intelligence, cardiovascular disease, diagnosis, machine learning, risk prediction

## Abstract

Cardiovascular diseases (CVDs) continue to be a major cause of death and morbidity throughout the world, and there is an increasing need for better diagnostic and predictive approaches. Existing methods do not fully reflect complex clinical interactions, and new computational methods possess greater capabilities. The limitations, including a lack of diversity in datasets, low generalizability, and limited interpretability, limit general use in the clinic. The review highlights the latest developments in artificial intelligence (AI) for diagnosing and predicting cardiovascular risk factors, with a focus on its clinical applications and current challenges. A literature review was conducted on machine learning (ML) and deep learning (DL) applications in imaging, electrocardiography (ECG), and predictive modeling. AI has been shown to improve diagnostic accuracy, facilitate earlier diagnosis, and enhance the ability to stratify disease risk compared with traditional methods. The seamless integration with wearable technologies ensures continuous monitoring and proactive management. While these developments help to ensure more accurate and individualized care, there are issues of validation, ethics, and integration. Moreover, integration of multi-modal data sources and real-time analytics enhances clinical decision-making and risk assessment. As technology continues to evolve, its scalability and applicability across various healthcare settings are expected to improve. In summary, AI has the potential to revolutionize cardiovascular care and enhance clinical outcomes by leveraging data-driven approaches.

## Introduction and background

Cardiovascular diseases (CVDs) remain major contributors to global morbidity and mortality and continue to impose a substantial burden on healthcare systems worldwide [1]. This burden has increased interest in artificial intelligence (AI)-enabled approaches that may support earlier diagnosis, more accurate risk prediction, and more efficient cardiovascular decision-making [2,3]. In cardiology, AI commonly includes machine learning (ML) and deep learning (DL), which can analyze complex clinical, imaging, electrocardiographic, and physiological data that may not be fully captured by conventional statistical or rule-based approaches [4-6].

The clinical interest in AI is partly driven by the need to interpret increasingly large and heterogeneous cardiovascular datasets, including electronic health records (EHRs), electrocardiograms (ECGs), imaging findings, wearable-device outputs, and routine clinical parameters [5,6]. Conventional cardiovascular risk models remain useful, but they may not fully account for nonlinear relationships among risk factors or interactions across multiple data sources [7]. ML-based and explainable AI approaches may address some of these limitations by identifying complex patterns and supporting more transparent risk estimation when externally validated [8].

Current evidence suggests that AI has potential applications across several cardiology domains, including cardiovascular diagnosis, risk prediction, electrocardiographic interpretation, imaging analysis, rhythm monitoring, and clinical decision support [6,9]. AI-based ECG tools have been studied for arrhythmia detection and heart failure risk prediction, and AI-supported echocardiography has been developed for automated assessment of cardiac structure and function [10,11]. In primary and ambulatory care settings, AI models are also being evaluated for cardiovascular risk prediction, although their clinical use requires careful assessment of calibration, validation, and applicability to real-world populations [12,13].

Despite rapid progress, several barriers limit the routine use of AI in cardiovascular care. These include restricted dataset diversity, retrospective model development, insufficient external validation, limited interpretability, algorithmic bias, privacy concerns, and difficulty integrating AI tools into clinical workflows [5,6,14]. These issues are clinically important because inaccurate or poorly generalizable models may produce unreliable risk estimates, reduce clinician trust, or worsen inequities across patient populations [5,12]. Prospective validation, transparent reporting, standardized implementation pathways, and regulatory oversight are therefore necessary before widespread clinical adoption [3,15].

Although several recent reviews have addressed AI in cardiology, many focus on individual domains such as imaging, ECG interpretation, digital health, or risk prediction. The specific need for this narrative review is to synthesize these related areas together and evaluate how AI may contribute to diagnosis, prognostication, monitoring, and implementation in cardiovascular practice. This review, therefore, aims to analyze the current state of AI applications in cardiology, specifically in diagnosis and risk prediction. It discusses ongoing research and developments, assesses clinical relevance, identifies limitations and research gaps, and provides an overview of future prospects for integrating AI into cardiovascular care.

Objective of the review

This review aims to analyze the current state of the art in the field of AI and its applications in cardiology, specifically in risk prediction and diagnosis. It discusses ongoing research and developments, assesses clinical relevance, and identifies limitations and research gaps. In addition, the review provides an overview of the prospects for integrating AI into cardiovascular care.

Literature search strategy

A narrative literature search was conducted to identify recent and clinically relevant evidence on AI applications in cardiology. This review was designed as a narrative review rather than a systematic review or meta-analysis; therefore, formal PRISMA (Preferred Reporting Items for Systematic Reviews and Meta-Analyses) flow reporting and quantitative pooling were not performed. PubMed/MEDLINE, Google Scholar, Scopus, Web of Science, and relevant cardiology and digital health sources were searched for articles published between 2017 and 2026 using combinations of terms including “artificial intelligence,” “machine learning,” “deep learning,” “cardiology,” “cardiovascular disease,” “risk prediction,” “electrocardiography,” “cardiovascular imaging,” “arrhythmia,” “heart failure,” “coronary artery disease,” “wearable devices,” “digital health,” “clinical validation,” “explainable AI,” “algorithmic bias,” “foundation model,” “transformer model,” “software as a medical device,” and “regulatory framework.” Representative Boolean search combinations included “artificial intelligence” AND “cardiology,” “machine learning” AND “cardiovascular risk prediction,” “deep learning” AND “electrocardiography,” “artificial intelligence” AND “cardiovascular imaging,” and “digital health” AND “cardiovascular disease prevention.”

Titles and abstracts were first screened for relevance to cardiovascular AI, followed by full-text assessment of articles considered potentially eligible. Priority was given to recent studies, high-impact reviews, clinical statements, and articles directly relevant to cardiovascular diagnosis, prognostication, monitoring, and implementation. Articles were considered eligible when they addressed AI-based cardiovascular applications or provided evidence on validation, interpretability, ethics, workflow integration, or regulatory considerations. Publications were excluded when they were not directly related to cardiovascular AI, did not support the cited claim, focused only on general computational methods without clinical cardiology relevance, were duplicate records, lacked accessible full text, or did not provide sufficient clinical context for the review objective.

Because the included literature covered heterogeneous study designs, data sources, AI tasks, outcome measures, and clinical settings, a quantitative synthesis or meta-analysis was not appropriate. No statistical summary method, meta-analysis, or meta-regression was performed because the included studies and reviews differed substantially in population, data type, AI model architecture, validation method, reported outcomes, and clinical application area. Therefore, pooled estimates, p-values, confidence intervals, heterogeneity statistics, and forest plots were not applicable to this narrative review. Formal risk-of-bias scoring using tools such as QUADAS-2 or ROBINS-I was not conducted because this was not a systematic review. Instead, the included literature was assessed narratively according to clinical relevance, methodological clarity, validation status, applicability to cardiovascular care, and direct alignment between each citation and the statement it supported. Additional statistical peer review was not required because no original statistical analysis, pooled quantitative comparison, or meta-analytic evidence synthesis was conducted. No review protocol was registered in PROSPERO, OSF, or other open-science registries because the manuscript was prepared as a narrative review. Findings were synthesized narratively by clinical application area, including ECG-based diagnosis, cardiovascular imaging, arrhythmia detection, risk prediction, heart failure, coronary artery disease (CAD), digital health, and implementation challenges.

## Review

Fundamentals of AI in cardiology

The development of AI in cardiology is supported by advances in ML-based cardiovascular risk prediction models, including approaches that compare established clinical models with ML-derived prediction tools [16]. These computational methods are increasingly applied across cardiology to support diagnostic assessment, risk stratification, workflow efficiency, and clinical decision-making [17]. In cardiology, AI applications are based on various data types, such as EHRs, imaging, ECG, and wearable devices, which can be combined to support more comprehensive cardiovascular assessment when data quality, interoperability, clinical context, governance, and external validation are adequately addressed [18].

Depending on the application, ML algorithms can be broadly classified into supervised, unsupervised, or reinforcement learning approaches and are used for cardiovascular research with varying applications [17]. The most common of these is supervised learning, in which models are trained on labeled data to predict outcomes such as disease presence, cardiovascular risk, or future clinical events [16,17]. In turn, unsupervised learning can identify hidden patterns or clusters within unlabeled clinical data, which may help characterize complex cardiovascular phenotypes [17]. Reinforcement learning is less commonly applied in routine cardiovascular practice but has potential relevance for adaptive decision-making and treatment optimization frameworks when supported by clear clinical governance and validation pathways [18].

DL uses multi-layer artificial neural networks (ANNs) and operates on high-dimensional data, such as medical images and waveform signals, as a specialized subset of ML with particular relevance to cardiovascular medicine [19]. These models have shown strong performance in tasks such as image analysis, ECG interpretation, and signal classification, making them relevant to several cardiology applications [19]. For instance, convolutional neural networks (CNNs) can be used to analyze cardiac imaging data, and recurrent neural networks (RNNs) can be used to analyze sequential data, such as ECGs. Due to their ability to automatically identify features, DL models can reduce dependence on manual feature engineering and improve performance in selected cardiovascular applications [19]. More recent AI development has also expanded toward transformer-based architectures and foundation-model approaches, particularly for large-scale ECG and multimodal cardiovascular data analysis. These models may improve transferability across diagnostic and predictive tasks, but their clinical value still requires transparent benchmarking, external validation, and prospective evaluation before routine adoption [20].

One of the key aspects of AI use in cardiology is the structured development and implementation of models, including data preprocessing, model training, validation, testing, and clinical evaluation [20]. Because inconsistencies or biases in the dataset can have a substantial impact on the model's performance and generalizability, data quality and representativeness remain critical requirements for reliable AI-based cardiovascular models [21]. For ECG-based and signal-based AI applications, model evaluation must account for diagnostic accuracy, validation on independent datasets, and the ability to generalize across recording conditions and patient populations [22,23]. Clinical implementation should also follow emerging governance principles, including transparency, prospective validation, post-deployment monitoring, regulatory oversight, and clear responsibility for clinician-facing decisions [18].

AI in ECG-based diagnostic applications

AI has contributed to cardiovascular diagnosis by enabling automated interpretation of ECG and other clinical data, including explainable decision models for cardiac disorder classification [24]. AI-enhanced electrocardiography has also been reviewed as a tool for improving the diagnosis and management of CVDs, particularly through automated analysis of ECG-derived patterns [25]. Models with an AI foundation, particularly those based on ML and DL, have shown potential to improve CVD prediction and diagnostic classification, although performance varies across datasets, methods, and validation approaches [26].

Among the most notable uses of AI in the diagnosis of CVDs is ECG interpretation. AI-based ECG approaches can support the detection and classification of cardiac disorders by analyzing electrical signal patterns that may be difficult to identify consistently using manual interpretation alone [24,27]. ML and DL methods have also been applied to CVD prediction tasks, supporting earlier identification of patients at risk when models are appropriately validated [27,28]. Hybrid and dataset-driven ML models have further demonstrated potential for cardiovascular risk classification, but their clinical utility depends on external validation and applicability to real-world populations [29,30].

Besides ECG analysis, AI has also been applied to structured dataset-based heart disease prediction, supporting automated classification and risk assessment in selected datasets [31]. This evidence should be interpreted as support for computational model development rather than direct proof of broad clinical implementation [32]. Overall, ECG-based and structured-data diagnostic models show promise for automated classification and early risk recognition, but the evidence remains heterogeneous across datasets, model architectures, and validation strategies [33]. For this reason, these tools should be interpreted as clinician-support systems rather than replacements for clinical assessment. Cardiovascular diagnosis is one of the areas where AI is finding important applications, as summarized in Table 1.

**Table 1:** Applications of Artificial Intelligence in Cardiovascular Diagnosis AI: Artificial Intelligence; CVD: Cardiovascular Disease; ML: Machine Learning; DL: Deep Learning; ECG: Electrocardiogram

Application Area	AI Technique Used	Clinical Function	Evidence/Clinical Relevance	Reference
ECG Analysis	Explainable AI/ML Models	Classification of ECG-based cardiac disorders	Supports interpretable ECG-based diagnostic decision-making	[24]
AI-Enhanced Electrocardiography	ML/DL Models	Diagnosis and management support for CVDs using ECG-derived data	Improves automated ECG interpretation and clinical decision support	[25]
CVD Prediction	ML/DL Models	Prediction and classification of cardiovascular disease risk	Supports risk assessment across diverse cardiovascular datasets	[26]
DL-Based CVD Prediction	DL-incorporated ML Models	CVD prediction using combined ML and DL approaches	Enhances predictive modeling for CVD risk assessment	[27]
Hybrid Risk Classification	Hybrid ML Models	Classification of cardiovascular risk using hybrid datasets	Improves risk prediction in selected datasets	[28]
Heart Disease Prediction	ML Techniques	Automated prediction of heart disease using structured clinical/computational features in selected datasets	Supports data-driven diagnostic and risk classification during model development	[29]

AI in ECG-based and rhythm diagnosis

AI has contributed to cardiovascular diagnosis by supporting the detection and management of arrhythmias through automated analysis of rhythm-related data [34]. In AF, ML methods have been studied for detection and management support, reflecting one of the major diagnostic applications of AI in cardiology [35]. Arrhythmia assessment is also clinically important in specific contexts such as PPCM, where timely recognition and management of rhythm disturbances may influence patient assessment and treatment planning [36].

ECG is one of the most important examples of AI's role in cardiovascular diagnostics. AI-enabled ECG and rhythm-monitoring approaches can support the detection of intermittent arrhythmias that may be missed by short-duration or symptom-dependent conventional monitoring strategies [37]. These approaches are particularly relevant for AF and other intermittent rhythm disorders, where continuous or repeated monitoring may improve diagnostic yield [35,38]. By analyzing ECG-derived rhythm patterns, AI models may help identify clinically relevant arrhythmias and support earlier diagnostic assessment when used with validated monitoring systems [34,39].

AI-based rhythm diagnosis is most clinically useful when it is integrated with an appropriate monitoring strategy, such as ambulatory ECG recording, wearable rhythm monitoring, or follow-up evaluation in patients with suspected intermittent arrhythmias [40]. These applications may improve detection opportunities, but their clinical reliability depends on device accuracy, algorithm validation, false-positive management, and clinician review [34,35,37].

**Figure 1:**
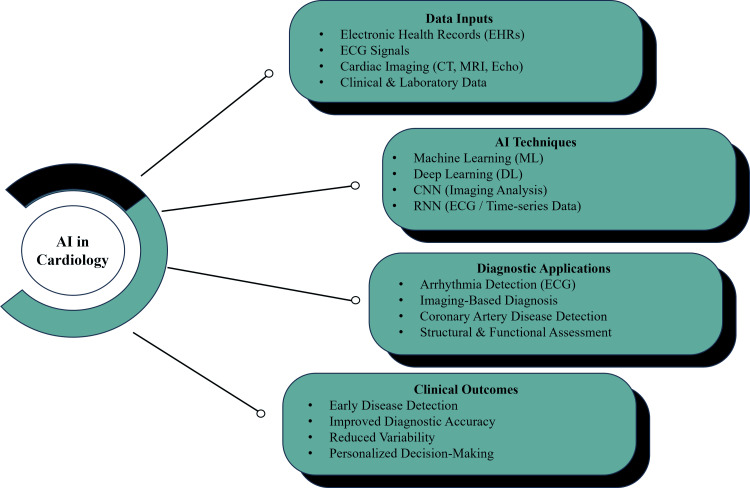
AI-Supported ECG-Based Cardiovascular Diagnostic Workflow The figure shows the AI-supported ECG-based diagnostic workflow, including data acquisition, preprocessing, model interpretation, clinician review, and diagnostic decision support. Image Credit: Anand Sekar G using Microsoft PowerPoint (Microsoft Corporation, Redmond, Washington).

Overall, AI-supported ECG and rhythm tools should be viewed as diagnostic-support technologies that may improve recognition of arrhythmias when validated appropriately, rather than as stand-alone replacements for standard cardiovascular evaluation. Figure 1 shows the AI-supported ECG-based diagnostic workflow.

AI in risk prediction and prognostication

The use of AI in cardiovascular risk prediction and prognostication is increasingly linked with digital health strategies for CVD prevention, where data-driven tools may support earlier risk identification, monitoring, and preventive care planning [41]. Digital health solutions can support cardiovascular prevention by enabling collection, interpretation, and use of patient-generated or routinely collected health data, although their effectiveness depends on appropriate validation and implementation [41]. These approaches are particularly relevant because cardiovascular risk prediction increasingly extends beyond static clinic-based assessment toward longitudinal monitoring and prevention-oriented care models [41].

ML and digital health technologies are also expected to contribute to future CVD prevention by supporting more individualized risk assessment, remote monitoring, and timely preventive interventions [42]. Compared with conventional risk assessment performed at isolated clinical encounters, AI-enabled digital approaches may allow repeated assessment of risk-related variables over time [42]. This longitudinal approach may help clinicians identify changes in cardiovascular risk profiles earlier and support more proactive prevention strategies [42].

Risk prediction models that are based on AI may incorporate data from digital health platforms, wearable devices, mHealth tools, clinical records, and patient-reported information to support cardiovascular prevention and management [43]. Such digital health approaches can assist in monitoring cardiovascular risk factors, supporting self-management, and improving communication between patients and healthcare professionals [43]. This broader integration of data may allow more personalized cardiovascular care, although implementation remains dependent on usability, data quality, accessibility, and integration into clinical workflows [43].

Another important consideration is whether digital health interventions for CVD management provide sufficient value for healthcare systems [44]. Evidence from cost-effectiveness research suggests that economic evaluation is necessary when assessing digital cardiovascular interventions, because clinical benefit, implementation cost, scalability, and long-term outcomes influence their practical adoption [44]. Therefore, AI-supported risk prediction and prognostication should be evaluated not only for predictive accuracy but also for clinical utility, cost-effectiveness, and sustainability in real-world care settings [44].

AI-enhanced electrocardiography also contributes to cardiovascular risk assessment and prognostication by extracting clinically relevant information from ECG signals that may support disease detection and management [45]. In CVD management, AI-enabled ECG tools have been applied to identify rhythm abnormalities and other ECG-derived markers that may inform diagnosis, monitoring, and risk evaluation [45]. These applications broaden the role of AI beyond traditional risk scores by linking physiological signal analysis with clinical decision-making [45].

Notwithstanding such benefits, several challenges remain in the application of AI and digital health tools to cardiovascular risk prediction and prognostication. These include the need for stronger clinical validation, reliable data integration, equitable access, patient privacy protection, cost-effectiveness assessment, and incorporation into routine clinical workflows [41-45]. These limitations should be considered when interpreting digital health-based risk prediction models, because strong predictive performance alone does not necessarily confirm clinical usefulness, equity, or implementation readiness. Table 2 illustrates some of the major applications.

**Table 2:** AI in Cardiovascular Risk Prediction AI: Artificial Intelligence; CVDs: Cardiovascular Diseases; ML: Machine Learning; DL: Deep Learning; RNNs: Recurrent Neural Networks; CNNs: Convolutional Neural Networks; MACE: Major Adverse Cardiovascular Events; MI: Myocardial Infarction; HF: Heart Failure

Application Area	AI Technique Used	Clinical Function	Evidence/Clinical Relevance	Reference
Digital Cardiovascular Risk Prevention	Digital Health and AI-Supported Tools	Supports CVD prevention through digital monitoring, risk assessment, and prevention-oriented data use	Facilitates earlier identification and management of cardiovascular risk factors when integrated with validated preventive-care pathways	[41]
Future Cardiovascular Prevention	ML and Digital Health Technologies	Supports individualized risk assessment, remote monitoring, and prevention planning	Enables more proactive and personalized cardiovascular prevention strategies but requires clinical validation before routine use	[42]
Digital Health-Based Risk Monitoring	mHealth and Wearable-Linked AI Approaches	Monitors cardiovascular risk factors and patient-generated health data over time	Supports self-management, longitudinal follow-up, and patient-clinician communication when data accuracy, usability, and response pathways are established	[43]
Economic Evaluation of Digital Interventions	Digital Health Intervention Assessment	Evaluates the cost-effectiveness of digital health tools used in CVD management	Supports decisions about scalability, sustainability, and healthcare system value based on implementation cost and long-term clinical benefit	[44]
AI-Enhanced Electrocardiographic Risk Assessment	AI-Enhanced ECG Models	Uses ECG-derived information to support CVD detection, monitoring, and management	Links physiological signal analysis with clinical decision-making and risk evaluation when externally validated and interpreted with clinical context	[45]

AI in cardiovascular imaging

AI has substantially influenced cardiovascular imaging, particularly through applications in image segmentation, automated quantification, diagnostic interpretation, and extraction of clinically relevant imaging features [30]. Conventional cardiovascular imaging interpretation often depends on expert visual assessment and manual measurements, which may be time-consuming and subject to inter-observer and intra-observer variability [30]. AI methods, especially DL methods, can support automated analysis of imaging data and may improve efficiency, reproducibility, and consistency in cardiovascular image interpretation [30].

CNNs have shown important applications in cardiovascular imaging modalities, including echocardiography, cardiac MRI, and CT [30]. These models are able to support segmentation of cardiac structures, quantification of ventricular function, and detection of structural or functional abnormalities [30]. AI-based imaging tools may assist in measuring parameters such as ventricular volumes, EF, and wall motion, which are relevant to the evaluation of heart failure, ischemic heart disease, and other structural cardiovascular conditions [30,31].

AI has found important applications in echocardiography and broader cardiovascular imaging by assisting automated functional assessment and improving workflow efficiency [31]. In echocardiography, AI-supported approaches may help evaluate cardiac structure and function, reduce measurement variability, and support more standardized image interpretation [31]. This allows for more reproducible diagnostic assessment and may assist clinicians in evaluating disease severity and progression when AI outputs are appropriately validated and clinically integrated [31].

Likewise, in CT and other cardiovascular imaging modalities, AI has been studied for automated image analysis, quantitative assessment, and improvement of workflow efficiency [32]. AI-based tools may assist imaging cardiologists by supporting image interpretation, reducing repetitive measurement tasks, and improving consistency across imaging workflows [32]. These developments are especially relevant in clinical settings where rapid, reproducible, and quantitatively reliable imaging interpretation is needed [32].

Risk stratification using imaging biomarkers is another significant use of AI in cardiovascular imaging. AI models may combine imaging-derived features with clinical data to support diagnostic evaluation, prognostic assessment, and individualized cardiovascular risk stratification [30,33]. This multidimensional analysis may contribute to more personalized treatment planning and patient management when supported by robust validation and appropriate clinical context [30,33].

Despite these advantages, several issues remain with the application of AI to cardiovascular imaging. Variability in imaging protocols, limited availability of high-quality annotated datasets, model interpretability, external validation, and generalizability across institutions remain important barriers to clinical implementation [30,32,33]. These barriers are especially relevant because imaging AI models may perform differently across scanners, acquisition protocols, institutions, and patient populations. Therefore, imaging-based AI tools should be evaluated not only for segmentation or measurement accuracy but also for reproducibility across imaging platforms, clinical workflow integration, and impact on diagnostic decision-making. Ethical, regulatory, and workflow-related considerations also need to be addressed to support the safe and effective clinical use of AI in cardiovascular imaging [32,33].

AI in arrhythmia detection and management

AI has demonstrated clinical relevance in cardiac arrhythmia detection and management by supporting automated analysis of rhythm data and assisting diagnostic and therapeutic decision-making [34]. Arrhythmias, including AF and ventricular rhythm disorders, are important cardiovascular conditions that require accurate detection, risk assessment, and appropriate management [34]. Traditional diagnostic procedures, such as periodic ECGs and Holter monitoring, may miss intermittent or asymptomatic rhythm disturbances when arrhythmic episodes do not occur during the recording period [37]. AI-based rhythm analysis may help address this limitation by supporting automated interpretation of ECG and rhythm-monitoring data when algorithms are validated in clinically relevant populations [34].

ML has been increasingly studied for the detection and management of AF, particularly through analysis of ECG and rhythm-monitoring signals [35]. These models can process large volumes of waveform data and may help identify rhythm abnormalities that are difficult to detect consistently through manual review alone [34,35]. Evidence-based monitoring strategies also emphasize the role of prolonged and novel monitoring approaches for intermittent arrhythmias, which may improve detection when conventional short-duration ECG testing is insufficient [37]. In specific clinical contexts, such as PPCM, careful detection and management of arrhythmias remain clinically important because rhythm disturbances may influence patient assessment and treatment planning [36].

Wearable devices, coupled with AI algorithms, have expanded opportunities for rhythm monitoring beyond traditional clinical settings, particularly by enabling repeated or prolonged assessment of cardiac rhythm [37]. Physiological data can be obtained over a prolonged period with the use of smartwatches, portable ECG monitors, and other ambulatory monitoring devices, which may help detect intermittent arrhythmias [37]. When combined with validated analytic algorithms, these devices may support earlier recognition of abnormal rhythms and improve referral or treatment decisions [35,37]. This approach is particularly relevant for AF screening, remote monitoring, and follow-up of patients with suspected intermittent rhythm disorders [35,37].

In addition to the detection component, AI is being explored for arrhythmia management to support risk assessment, treatment planning, and interpretation of patient-specific rhythm data [34]. ML models may incorporate clinical history, ECG features, imaging findings, and electrophysiological information to assist in estimating recurrence risk or guiding individualized management strategies [34,35]. Such applications may support decisions related to pharmacologic therapy, follow-up monitoring, and invasive rhythm-control strategies, although clinical implementation requires appropriate validation [34,35].

**Figure 2:**
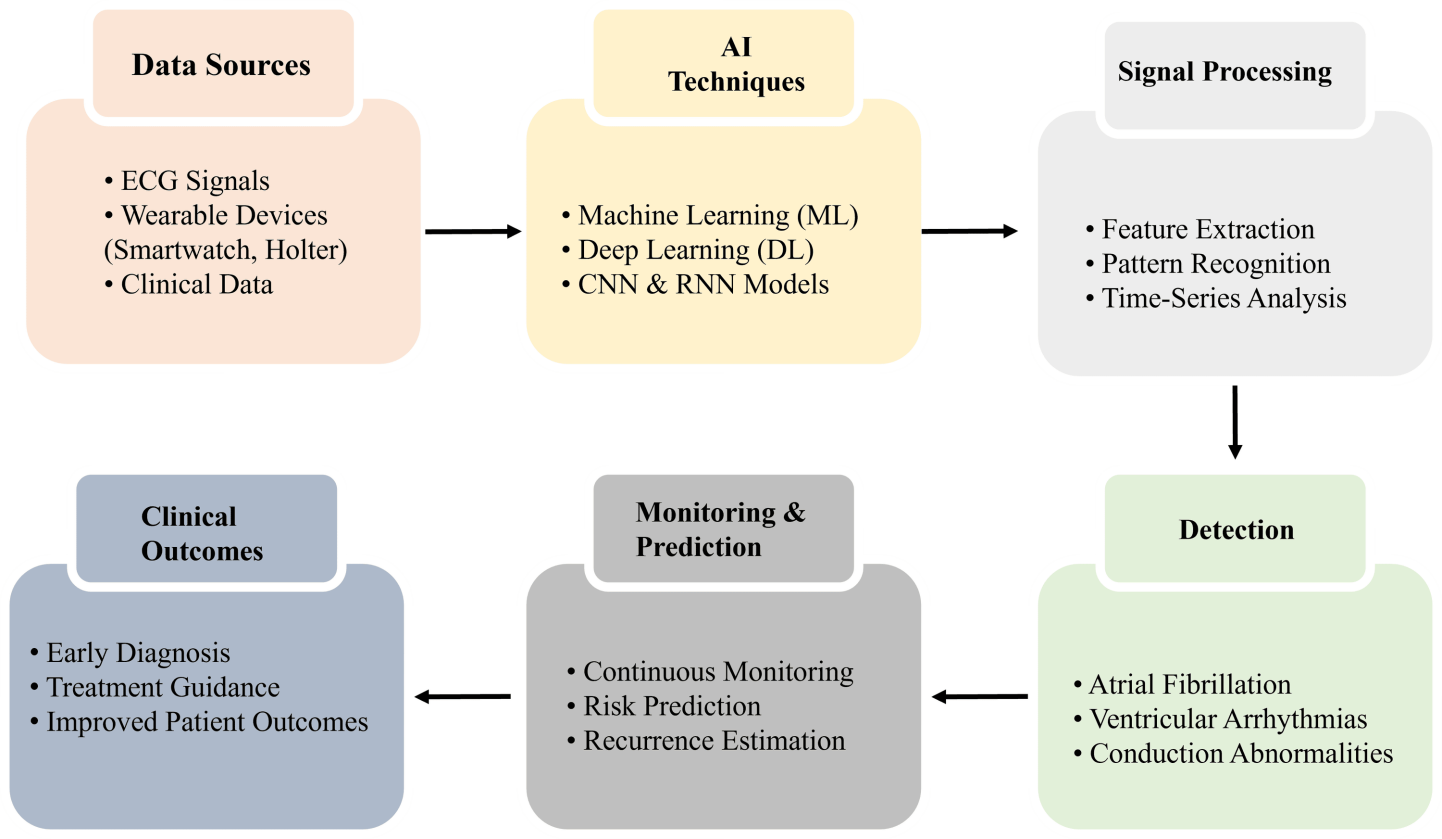
AI-Supported Arrhythmia Detection and Management Pathway The figure shows the AI-supported arrhythmia detection and management pathway, including rhythm data acquisition, algorithmic analysis, abnormal rhythm identification, clinician confirmation, and follow-up decision-making. Image Credit: Anand Sekar G using Microsoft PowerPoint (Microsoft Corporation, Redmond, Washington).

AI may also provide EP procedural support by helping interpret arrhythmia mechanisms, supporting mapping strategies, and assisting procedural planning in selected settings [34]. AI-assisted approaches may contribute to more precise identification of arrhythmogenic substrates, but their impact on procedural outcomes and recurrence reduction requires further clinical validation [34]. Despite these developments, broad implementation of AI in arrhythmia management remains difficult. Data quality, algorithm transparency, device accuracy, false-positive alerts, external validation, and integration into clinical workflows remain important barriers to broad clinical adoption [34,35,37]. Overall, AI-supported arrhythmia tools may improve rhythm detection and management support, but their clinical use should remain linked to validated devices, appropriate monitoring duration, clinician interpretation, and evidence of improved patient-relevant outcomes. Figure 2 depicts the AI-driven ECG analysis workflow and clinical impact.

AI in heart failure and CAD

AI has emerged as an important area of investigation in HF care, with potential applications in diagnosis, risk stratification, prognostic assessment, and treatment decision support [38]. HF is a significant source of morbidity and mortality, and its clinical presentation is often heterogeneous, requiring integration of clinical findings, imaging data, biomarkers, comorbidities, and longitudinal patient information [38]. AI methods may help address this complexity by identifying patterns across multidimensional datasets and supporting more individualized assessment of HF phenotypes and outcomes [38].

The application of ML algorithms in HF has been explored for diagnosis, prediction, patient phenotyping, and clinical risk assessment using routinely collected clinical and diagnostic data [38]. These models may incorporate demographic variables, laboratory findings, imaging parameters, comorbidities, and other clinical features to support earlier recognition of cardiac dysfunction and adverse outcome risk [38]. Such approaches may assist clinicians in identifying patients at higher risk of deterioration, hospitalization, or mortality, although their clinical use depends on external validation and integration into care pathways [38].

AI can also assist in HF diagnosis through computational analysis of clinical and diagnostic data used to identify HF-related patterns [39]. Algorithms can support recognition of HF by analyzing variables related to cardiac function, patient characteristics, and diagnostic profiles [39]. These tools may complement clinician assessment by improving diagnostic support, but they should not replace standard clinical evaluation, imaging interpretation, or guideline-based management [39].

The potential for improving the diagnostic and prognostic value of CAD has also been explored in selected AI-assisted intracoronary imaging contexts [40]. In patients with Takotsubo cardiomyopathy and CAD, AI and OCT have been used to evaluate in-hospital HF, illustrating how AI-supported intracoronary imaging analysis may contribute to selected CAD-related clinical assessments [40]. This evidence supports a cautious interpretation: AI may assist CAD-related evaluation in specific imaging and clinical contexts, but broader claims about CAD diagnosis, plaque characterization, or stenosis assessment require support from dedicated CAD imaging studies [40].

AI-based clinical decision support may help synthesize patient data and support individualized management in HF and selected CAD-related settings [38,40]. These advantages remain limited by data heterogeneity, incomplete external validation, interpretability concerns, and the need for prospective evidence demonstrating clinical utility [38,39]. Overall, AI has potential to improve aspects of cardiovascular care through earlier recognition, individualized assessment, and decision support, but routine implementation requires robust validation and careful integration into clinical workflows [38-40]. For heart failure and CAD, future clinical adoption should depend on disease-specific validation, integration with guideline-based care, and evidence that AI-supported decisions improve patient-relevant outcomes rather than only model performance. Some of the important applications are displayed in Table 3.

**Table 3:** AI in Heart Failure and Coronary Artery Disease AI: Artificial Intelligence; ML: Machine Learning; DL: Deep Learning; RNNs: Recurrent Neural Networks; CAD: Coronary Artery Disease; CCTA: Coronary Computed Tomography Angiography

Application Area	AI Technique Used	Clinical Function	Evidence/Clinical Relevance	Reference
HF Diagnosis and Risk Assessment	ML/AI-Based Models	Supports diagnosis, phenotyping, and risk assessment in HF using clinical and diagnostic data	May improve individualized assessment of HF patients when externally validated in clinically relevant populations	[38]
HF Prognostication	ML Models	Identifies patterns associated with deterioration, hospitalization, mortality, and adverse outcomes	Supports risk stratification and treatment planning when validated against clinically meaningful outcomes	[38]
HF Diagnostic Support	AI-Based Diagnostic Models	Analyzes clinical and diagnostic variables related to HF recognition	May complement clinician assessment and improve diagnostic support without replacing standard clinical evaluation	[39]
Multi-parameter HF Assessment	AI/ML Models	Integrates demographic, laboratory, imaging, comorbidity, and clinical information	Supports more comprehensive evaluation of cardiac dysfunction and patient risk across multimodal clinical data	[38,39]
CAD-Related AI Imaging Assessment	AI-Assisted Imaging/OCT Analysis	Evaluates selected CAD-related clinical contexts, including in-hospital HF in patients with Takotsubo cardiomyopathy and CAD	Illustrates the potential role of AI-supported intracoronary imaging analysis in selected CAD-related settings; it should not be interpreted as broad evidence for general CAD diagnosis	[40]
Clinical Decision Support	AI-Based Decision Support Models	Synthesizes patient-specific data to support individualized assessment and management planning	May assist clinical decision-making in HF and selected CAD-related contexts when integrated with guideline-based care and clinician oversight	[38,40]
Implementation Considerations	Validated AI Frameworks	Addresses the need for external validation, interpretability, and integration into clinical workflows	Supports cautious and evidence-based adoption in routine cardiovascular care with prospective validation and workflow assessment	[38-40]

Integration with digital health and wearable technologies

AI, digital health systems, and wearable devices have expanded opportunities for CVD prevention by supporting remote monitoring, patient engagement, and collection of longitudinal health data [41]. Digital health solutions for CVD prevention include tools that may assist risk-factor monitoring, lifestyle support, clinical follow-up, and data-driven preventive care [41].

ML and digital health technologies are increasingly viewed as part of the future of cardiovascular prevention, particularly because they may support individualized risk assessment and earlier preventive intervention [42]. Smartwatches and handheld ECG devices can be used to monitor physiological variables, including heart rhythm and activity-related measures, and may support early recognition of abnormal cardiovascular patterns when linked to validated clinical workflows [42].

Wearables that use AI are especially useful in the detection of arrhythmias, such as AF, because intermittent rhythm disturbances may not be captured during routine clinic-based assessments [42]. ML algorithms can process longitudinal physiological data and may generate alerts for abnormal patterns, supporting remote monitoring and timely clinical review [42]. These tools may reduce some monitoring burdens by extending observation beyond the clinic, but their clinical benefit depends on accuracy, validation, and appropriate response pathways [42].

mHealth apps also supplement these technologies through the provision of platforms for tracking cardiovascular health metrics, supporting patient education, and facilitating self-management [43]. AI can be used to analyze longitudinal data in order to support risk awareness, preventive behaviors, and follow-up in CVD prevention and management [43]. Additionally, AI is involved in preventive cardiology, in which it may support personalized feedback and lifestyle-related recommendations to reduce cardiovascular risk [43].

Cost-effectiveness, scalability, and health-system value are important considerations for digital health interventions in CVD management [44]. Even when digital tools show clinical promise, their adoption depends on whether they can be implemented sustainably, equitably, and efficiently in real-world healthcare systems [44].

In general, AI-enabled digital health systems can provide continuous or repeated monitoring, early screening support, and individualized cardiovascular care pathways [45]. AI-enhanced electrocardiography is particularly relevant to this integration because ECG-derived data can support CVD detection, monitoring, and management [45]. These merits exist, although data accuracy, privacy, interoperability, equity of access, and integration with clinical workflows remain barriers to widespread adoption [41,44,45]. Continuous data collection alone does not ensure clinical accuracy, equitable access, or improved patient outcomes; therefore, wearable- and mHealth-linked AI tools should be implemented only with appropriate validation, privacy protection, interoperability planning, and defined clinical response pathways.

Limitations and future directions

There are certain limitations in this narrative review. The results are mostly based on related past studies, most of which depend on retrospective data and might not completely reflect actual clinical conditions. Diversity in study design, databases, and evaluation measures across studies also restricts comparability and generalizability. Moreover, due to the rapid development of AI technologies, there is a risk that some current developments are yet to be fully reflected.

In a more general sense, limitations such as restricted data diversity, risk of algorithmic bias, and limited model explainability remain barriers to clinical adoption. Issues concerning data privacy, security, and regulatory approval also make implementation in everyday practice more difficult. Future research should prioritize large, multicenter, prospective studies to validate AI models across diverse population groups. Such directions, including greater focus on explainable AI, standardized frameworks, and the incorporation of multi-modal data, will help increase reliability and clinical applicability, while regulatory guidance will support safe and effective implementation of AI in cardiology. Future work should also include clinically meaningful outcome assessment, post-deployment performance monitoring, health equity evaluation, and assessment of model safety across different healthcare settings to ensure that AI tools remain reliable after implementation.

## Conclusions

This review concludes that AI has become an essential development in cardiology, improving the precision and efficiency of cardiovascular diagnosis and risk prediction. The combination of ML and DL with various clinical data, imaging methods, and wearable devices has supported earlier recognition and management of CVDs and provided more accurate prognostic evaluation. Such developments justify a transition to personalized medicine, in which treatment plans are determined based on the unique features of a patient. Applications in arrhythmia detection, cardiovascular imaging, HF, and CAD have shown substantial clinical potential for decision-making and patient outcome improvement. Also, the use of digital health tools may support longitudinal monitoring and preventive disease management; however, issues such as data heterogeneity, lack of interpretability, and barriers to clinical integration remain. These problems need to be addressed through standardized frameworks, strict validation, and ethical implementation. As research and development progress, AI may become an even more central part of cardiovascular care. Enhancing data infrastructure and model transparency will also facilitate its implementation into daily clinical practice.
